# Independent *S*-Locus Mutations Caused Self-Fertility in *Arabidopsis thaliana*

**DOI:** 10.1371/journal.pgen.1000426

**Published:** 2009-03-20

**Authors:** Nathan A. Boggs, June B. Nasrallah, Mikhail E. Nasrallah

**Affiliations:** Department of Plant Biology, Cornell University, Ithaca, New York, United States of America; The University of North Carolina at Chapel Hill, United States of America

## Abstract

A common yet poorly understood evolutionary transition among flowering plants is a switch from outbreeding to an inbreeding mode of mating. The model plant *Arabidopsis thaliana* evolved to an inbreeding state through the loss of self-incompatibility, a pollen-rejection system in which pollen recognition by the stigma is determined by tightly linked and co-evolving alleles of the S-locus receptor kinase (SRK) and its S-locus cysteine-rich ligand (SCR). Transformation of *A. thaliana*, with a functional *AlSRKb-SCRb* gene pair from its outcrossing relative *A. lyrata*, demonstrated that *A. thaliana* accessions harbor different sets of cryptic self-fertility–promoting mutations, not only in *S-*locus genes, but also in other loci required for self-incompatibility. However, it is still not known how many times and in what manner the switch to self-fertility occurred in the *A. thaliana* lineage. Here, we report on our identification of four accessions that are reverted to full self-incompatibility by transformation with *AlSRKb-SCRb*, bringing to five the number of accessions in which self-fertility is due to, and was likely caused by, *S-*locus inactivation. Analysis of *S-*haplotype organization reveals that inter-haplotypic recombination events, rearrangements, and deletions have restructured the *S* locus and its genes in these accessions. We also perform a Quantitative Trait Loci (QTL) analysis to identify modifier loci associated with self-fertility in the Col-0 reference accession, which cannot be reverted to full self-incompatibility. Our results indicate that the transition to inbreeding occurred by at least two, and possibly more, independent *S-*locus mutations, and identify a novel unstable modifier locus that contributes to self-fertility in Col-0.

## Author Summary

The mating system adopted by a species has a profound influence on extent of polymorphism, population structure, and evolutionary potential. In flowering plants, the switch from outbreeding to inbreeding has occurred repeatedly, yet little is known about the underlying genetic events. This is true even for the model species *A. thaliana*, a highly self-fertile member of the crucifer family. In this family, outbreeding is enforced by a self-incompatibility system controlled by the *S* locus, which involves the recognition of pollen by the stigma to prevent self-fertilization and familial inbreeding. We recently demonstrated that *A. thaliana* accessions may be reverted to full or partial self-incompatibility by transformation with *S-*locus genes isolated from its close self-incompatible relative *A. lyrata.* Despite much recent debate, however, we still do not know how *A. thaliana* became self-fertile. Here, we use our recently established *A. thaliana* transgenic self-incompatible experimental model to address these issues. Analysis of the *S* locus in accessions that can be reverted to full self-incompatibility demonstrates that self-fertility in *A. thaliana* arose by at least two independent *S-*locus mutations. Furthermore, analysis of an accession that expresses only partial self-incompatibility shows that self-fertility is associated with an unstable allele at a locus unlinked to the *S* locus.

## Introduction

Sexual reproduction may have evolved because it can combine different sequence variants through recombination [1] and because it can remove deleterious mutations linked to advantageous ones [2],[3]. However, approximately 20% of flowering plants are self-fertilizing and engage in sexual reproduction without obtaining either of these benefits [4]. It has been proposed that inbreeding plant lineages represent evolutionary “dead ends” [5] that evolved from outbreeding ancestors [4]–[6]. In this view, mating system switches from an outbreeding to inbreeding mode may have been selected for by pollinator scarcity or population bottlenecks [7], with inbreeding providing the benefits of reproductive assurance and increased potential for colonization, and in some cases possibly representing a survival mechanism used as a last resort to perpetuate a species. Because the outbreeding mode of mating is typically associated with the accumulation of recessive deleterious alleles that cause inbreeding depression, self-fertile taxa can only become established if this genetic load is purged. Theoretical models of the evolution of selfing have shown that inbreeding depression can indeed be overcome and selfing alleles can spread when the advantage of reproductive assurance outweighs the reduction of fitness [8]. However, mechanistic studies of switches from outbreeding to self-fertility have rarely been performed, and the genetic basis of these switches is poorly understood.

In the crucifer (Brassicaceae) family, switches to inbreeding have occurred frequently and entailed loss of self-incompatibility (SI). Self-incompatibility is a barrier to self-fertilization that is determined by variants of a single highly polymorphic locus, called the “*S* locus”. In self-incompatible plants, pollen is prevented from hydrating, germinating, and producing pollen tubes at the stigma surface if the same “*S-*locus” variant is expressed in pollen and stigma, whether these structures are located within the same flower or derived from different flowers on the same plant or different plants (for recent review, see [9]). As a result, self-incompatible plants are largely but not completely self-sterile, and autonomous seed set is typically less than 5% that set by self-compatible plants. In all self-incompatible crucifer species investigated to date, the “*S* locus” is not a single gene, but rather consists of two polymorphic genes, allelic forms of which together constitute a unique *S-*locus haplotype (hereafter *S* haplotype) that defines a unique recognition specificity. One gene encodes the S-locus Receptor Kinase (SRK) [10] and the second gene encodes the small S-locus Cysteine-Rich protein (SCR), which is the ligand for SRK. *SRK* is expressed in stigma epidermal cells, and its product is anchored via a single transmembrane domain in the plasma membrane of these cells. *SCR* is expressed in the anther tapetum, a cell layer that lines the sacs in which pollen grains develop, from which its SCR product is secreted and becomes incorporated into the outer pollen coat [11]. SCR proteins are delivered to the stigma surface upon pollen-stigma contact, but an SCR will bind to the extracellular domain of SRK and activate its cytoplasmic kinase domain, thus triggering the SI response, only if the SRK and the SCR proteins are encoded by the same *S*-locus haplotype [12],[13], i.e. when stigmas are pollinated with pollen derived from the same plant or from plants expressing the same *S* haplotype.

In view of this *S* haplotype-specific interaction, recombination events that disrupt the genetic linkage of matched *SRK* and *SCR* alleles will cause loss of SI. Consequently, there is strong selection for maintaining the tight linkage of these genes. Recombinants between *SRK* and *SCR* are rare in self-incompatible plants, either because self-compatible genotypes that might arise do not persist in nature (due to their genetic load) or because recombination is actively suppressed in the *S-*locus region [14]–[17]. Similar to other genomic regions exhibiting low effective recombination rates [18]–[20], the *S* haplotypes of self-incompatible *Brassica* and *A. lyrata* strains have been shown to accumulate haplotype-specific sequences due to divergent evolutionary trajectories and independent degeneration of non-coding sequences, and these features no doubt limit recombination in the region [14], [17], [21]–[23].

The model dicot plant *Arabidopsis thaliana* is a highly self-fertile crucifer that is thought to have had a self-incompatible ancestor based upon phylogenetic inference [24] and rescue of the SI trait by transgenic complementation with a functional *SRK-SCR* allelic pair from its close self-incompatible relative *A. lyrata* [25],[26]. However, despite several recent studies and much debate [27]–[31], the nature and number of mutational events that caused the switch to self-fertility in the *A. thaliana* lineage have not been established. Consistent with the expectation that selective pressures for maintaining the integrity of the *S* locus and its genes would be relaxed subsequent to the switch to self-fertility, all *A. thaliana* accessions analyzed to date harbor a non-functional *S* locus, referred to as pseudo-*S* (*ΨS*), which carries inactivating mutations in the *SRK* and/or *SCR* genes [23]. Analysis of *SRK* and *SCR* sequence divergence in various accessions identified three distinct *ΨS* haplotypes, designated *ΨSA*, *ΨSB*, and *ΨSC* [23],[28],[29],[32]. These three *A. thaliana ΨS* haplotypes are inferred to be orthologous, respectively, to the *S37*, *S16,* and *S36* haplotypes of *A. lyrata.* This conclusion is based on the observation that *SRK* or *SCR* sequences in the *A. lyrata S37*, *S16,* and *S36* haplotypes share much higher sequence similarity with the *ΨSRK* or *ΨSCR* sequences of the *A. thaliana ΨSA*, *ΨSB*, and *ΨSC* haplotypes, respectively, than with other *A. lyrata S* haplotypes [30].

Despite clear evidence for inactivating mutations in the *SRK* or *SCR* sequences of many *A. thaliana* accessions [23],[27],[28], it is not possible to conclude that inactivation of the *S* locus was the primary cause of the switch to self-fertility in all *A. thaliana* populations. Indeed, the species also harbors mutations at other genes required for SI, as indicated by differences among accessions in the ability to express SI upon transformation with *A. lyrata SRKb-SCRb* (*AlSRKb-SCRb*) genes [26],[33]. Among seven accessions analyzed by inter-specific complementation experiments, only C24 yielded a developmentally-stable SI response identical to that of *A. lyrata Sb* plants (<5 pollen tubes/self-pollinated stigma at all stages of stigma development), demonstrating unequivocally that a non-functional *S* locus is the only cause of self-fertility in this accession [26],[27]. By contrast, in other accessions, SI was transient [starting strong (<5 pollen tubes/self-pollinated stigma) in young flower buds, and later breaking down (>100 pollen tubes/self-pollinated stigma) in older flower buds and flowers], weak (25–50 pollen tubes per self-pollinated stigma), or absent (large numbers of pollen tubes/self-pollinated stigma at all stages of stigma development, similar to wild type untransformed *A. thaliana*). These phenotypes indicate the presence of mutations not only at the *S* locus, but also at “SI modifier” loci required for SI [26],[33]. Indeed, one such SI modifier was identified in a cross between a C24::*AlSRKb-SCRb* transformant, which expresses a robust and developmentally-stable SI response, and a plant from the *ΨSA*-containing RLD accession, which expresses transient SI [33]. Molecular genetic analysis of this cross determined that transient SI is associated with reduced *SRK* transcript levels in older flowers caused by sequences upstream of the Col-0 allele of *PUB8* (*P*lant *U-B*ox 8), a gene tightly-linked to the *S* locus [33].

A comprehensive understanding of the switch to self-fertility in *A. thaliana* requires analysis of the *S* locus and of SI modifier loci, because any of these loci might have been targets of selection for self-fertility. Accordingly, we used a two-pronged approach to elucidate the genetic events that accompanied the evolution of self-fertility in *A. thaliana*. Firstly, we transformed several *A. thaliana* accessions with the *AlSRKb-SCRb* genes in an attempt to identify accessions like C24, which express a robust and developmentally-stable SI response, and would therefore harbor mutations at the *S* locus but not at SI modifier loci. We reasoned that only in such accessions might it be possible to determine if the transition from outbreeding to inbreeding in *A. thaliana* occurred by a single mutational event or by multiple independent events. Secondly, we performed a Quantitative Trait Loci (QTL) analysis of SI modifier loci that differentiate *AlSRKb-SCRb* transformants of the reference Columbia (Col-0) accession, which express transient SI, from those of the C24 accession.

## Results

### Identification of *A. thaliana* Accessions That Express a Developmentally Stable Transgenic SI Response

To identify additional *A. thaliana* accessions, which, like C24, might express a robust and developmentally-stable SI phenotype, we transformed several previously-untested accessions with *AlSRKb-SCRb.* In selecting accessions for transformation, we excluded accessions that carry the *ΨSA* haplotype [27] and its closely-linked *PUB8* allele previously associated with transient SI [33], because *AlSRKb-SCRb* transformants of these accessions are not expected to express stable SI. For each selected accession, independent *AlSRKb-SCRb* transformants were generated and tested for SI by pollination assays at different stages of stigma development (Table 1). *AlSRKb-SCRb* transformants of four accessions, Sha, Kas-2, Hodja, and Cvi-0, were found to express a developmentally-stable SI phenotype identical to that observed in C24::*AlSRKb-SCRb* transformants and in *A. lyrata Sb* plants [26]: immature floral buds were self-compatible, and strong inhibition of self pollen was first detected in stage-13 buds and persisted in older flowers. In addition, there was very little seed set on these plants, either by open pollination (Table 1) or following manual self-pollination of mature floral buds and flowers. Significantly, these self-incompatible phenotypes are stably transmitted to subsequent transgenic generations, as determined by analysis of pollination phenotype over 20 generations in C24, 10 generations in Sha, and two generations in each of Cvi-0, Kas-2, and Hodja.

**Table 1:** *A. thaliana* accessions that express developmentally-stable transgenic SI.

Transgenic Strain	T1 Plants	Floral Stage a	Self-Pollination Phenotype^b^	Seed Set c
Hodja::*AlSRKb-SCRb*	2/2	floral bud 13	SI	
		open flower	SI	258±34
Sha::*AlSRKb-SCRb*	8/10	floral bud 13	SI	
		open flower	SI	219±25
Kas-2::*AlSRKb-SCRb*	5/7	floral bud 13	SI	
		open flower	SI	346±25
Cvi-0::*AlSRKb-SCRb* d	9/10	floral bud 13	SI	
		open flower	SI	63±11
C24::*AlSRKb-SCRb* e	11/18	floral bud 13	SI	
		open flower	SI	54±7
Col-0::*AlSRKb-SCRb* e	7/12	floral bud 13	SI	
		open flower	SC	∼10,000
untransformed	-	floral bud 13	SC	
		open flower	SC	∼10,000

a Floral bud 13: Stage-13 of flower development according to [54].

b SI: self-incompatible; SC: self-compatible.

c Number of seeds produced per plant over its lifetime by open pollination. The number±SD is based on averaging the number of seed produced by three plants in each group (two plants for Hodja). With the exception of Col-0::*AlSRKb-SCRb* transformants, which set as much seed as untransformed plants, *AlSRKb-SCRb* transformants of the other accessions shown produced very low seed set. This low seed set reflects a minor degree of leakiness of the SI trait equivalent to that observed with some naturally self-incompatible *A. lyrata* genotypes.

d Some of these plants were generated by transformation with *AlSRKb-SCRb* in a pCAMBIA1300 vector and selection on hygromycin.

e Similar results were previously reported for these accessions [26].

### Analysis of *ΨS*-Locus Haplotypes

Our successful complementation of the Sha, Kas-2, Hodja, and Cvi-0 accessions suggests that self-fertility in these accessions is due to a non-functional *S* locus, as in the C24 accession. It is therefore of interest to determine if the *ΨS*-haplotypes in these five accessions are the same or different (i.e. are likely to be derived from the same ancestral mutant *ΨS*-haplotype or from independently-derived ancestral *ΨS*-haplotypes).

At present, detailed descriptions are available only for the Col-0, C24, and Cvi-0 *ΨS* haplotypes. The Col-0 reference accession was shown to harbor a *ΨSA* haplotype containing aberrant *SRK* and *SCR* sequences. Its *ΨSRKA* allele contains a frameshift mutation that introduces a premature stop codon within the fourth of seven exons found in *SRK* genes. Its *SCR* sequences consist of several truncated *ΨSCR* sequences, the longest of which is designated *ΨSCR1* [23]. In contrast, the C24 *ΨS* haplotype was shown to have been produced by recombination between *ΨSA* and *ΨSC* haplotypes [27]: it contains rearranged remnants of *ΨSRKA* exon 1 [which encodes the *SRK* extracellular domain (ΨeSRK)], a truncated version of *ΨSRKC* consisting of exon 7, and two copies of *ARK3* (At4g21380), a polymorphic gene located at one flank of the *S* locus in *Arabidopsis* species [23]: one copy consists of an *ARK3^SC^* allele characteristic of *ΨSC* haplotypes located at its normal location and an additional chimeric *ARK3* copy located between the *ΨSRKA* and *ΨSRKC* sequences, which resulted from recombination between an *ARK3^SC^* allele and an *ARK3^SA^* allele characteristic of *ΨSA* haplotypes. As for the Cvi-0*ΨSB* haplotype, its complete DNA sequence (accession number EF637083 [29],[32]) revealed the presence of a *ΨSRKB* allele containing a splice-site mutation at the end of intron 2 [28] and a convergently-oriented *ΨSCRB* allele lacking obvious inactivating mutations [29],[32].

#### Distinct *S-*locus structures in accessions exhibiting a developmentally-stable transgenic SI phenotype

**Figure 1:**
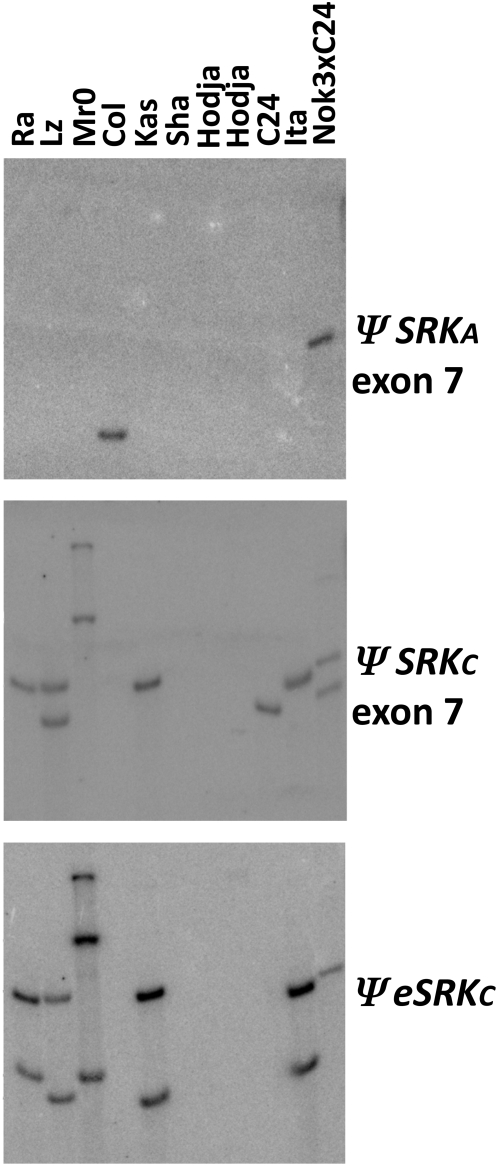
DNA gel blot analysis of *A. thaliana ΨS*-locus sequences in various *A. thaliana* accessions. A blot of EcoRI-digested genomic DNA was probed (as indicated underneath the blots) sequentially with *ΨSRKA* exon 7 derived from the Col-0 accession, *ΨSRKC* exon 7 derived from the Ita-0 accession, and the extracellular domain of *ΨSRKC* (*ΨeSRKC*) also derived from the Ita-0 accession. A Nok-3 x C24 hybrid was used to assess Nok-3 *S*-locus polymorphisms because at the time of producing the blot, there was no pure Nok-3 DNA available. Nok-3 was determined to have sequences corresponding to *ΨSRKC* exon 7, similar to C24, because when probed with this fragment, the Nok-3 x C24 hybrid exhibits two hybridizing bands, whereas C24 exhibits only one.

Previous studies had shown that the Kas-2 *ΨS* haplotype contains a full-length *ΨSRKC* sequence with no obvious inactivating mutations [27],[28], and that the Sha and Hodja *ΨS* haplotypes contain similar *ΨSRKA* sequences based on identical DNA gel blot hybridization patterns with a *ΨSRKA* probe [27]. To obtain a more detailed description of the *ΨS* haplotypes of Kas-2, Sha, and Hodja, we assayed these accessions, along with Cvi-0, C24, Col-0 and several other accessions included for comparison, using markers known to be diagnostic of each of the *ΨSA, ΨSB*, and *ΨSC* haplotypes [27]. The presence and integrity of *ΨSRKA*, *ΨSRKB*, and *ΨSRKC* sequences, as well as the occurrence of *SA*-*SC* inter-haplotypic recombination similar to that observed in the C24 *S* haplotype [27], were assayed by DNA gel blot analysis (Figure 1) using probes corresponding to exon 1 of *ΨSRKA* , *ΨSRKB*, and *ΨSRKC* (i.e. *ΨeSRK* s), and to exon 7 of *ΨSRKA* and *ΨSRKC*. Furthermore, PCR was used to screen for the chimeric copy of *ARK3* found in the C24 *S* haplotype, and to assay for the presence/absence of a first-intron deletion in *ARK3* as a means to differentiate the *ARK3^SC^* allele, which has the deletion, from the *ARK3^SA^* allele, which lacks the deletion. Because recombination between highly-diverged *ΨS* haplotypes is infrequent [see Discussion], these *ARK3* polymorphisms can differentiate between *ΨSA* and *ΨSC* haplotypes [27].

**Figure 2:**
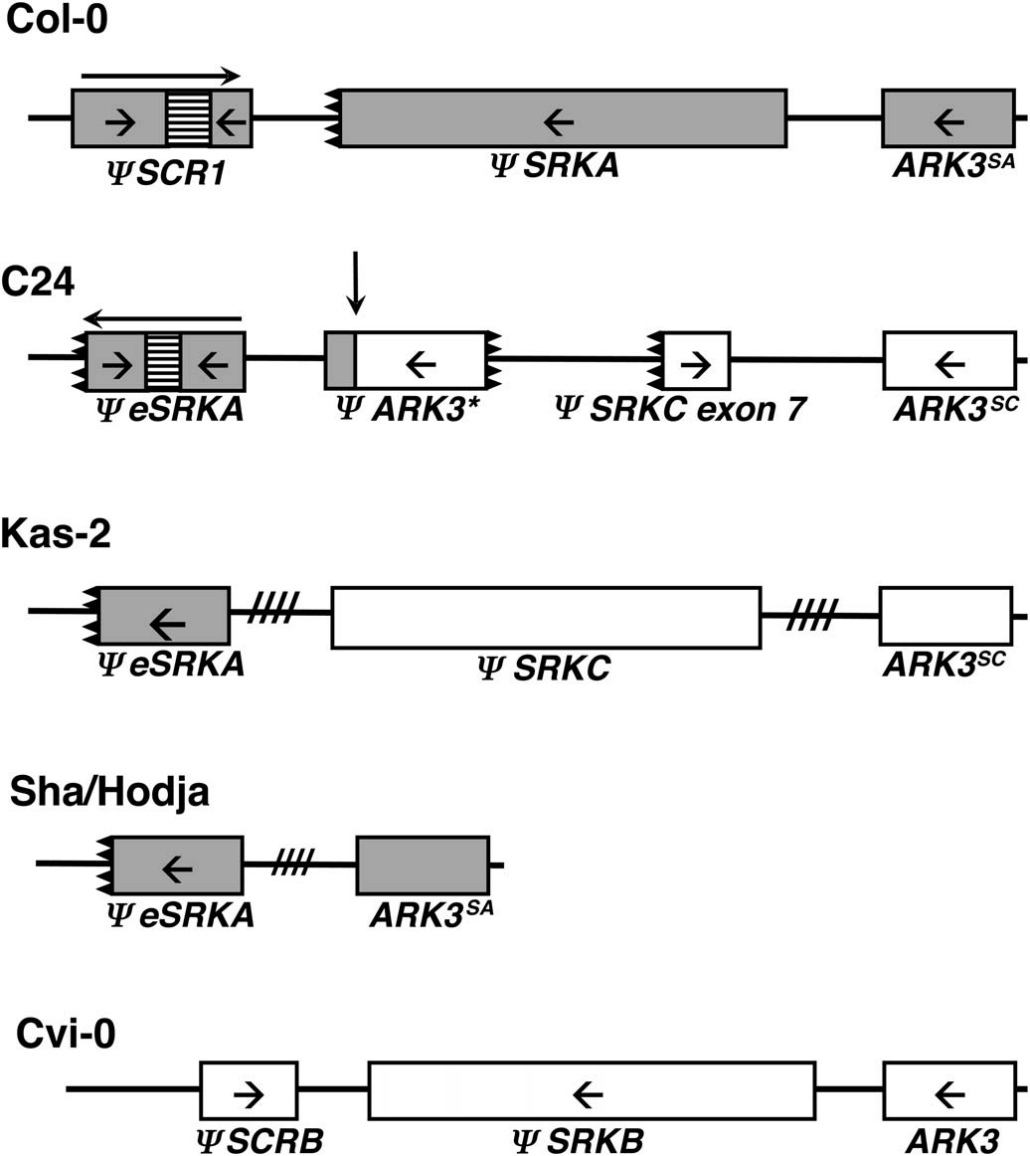
*ΨS*-locus structure in Col-0 and accessions that express a developmentally-stable transgenic SI response. The *ΨS*-locus genes and gene fragments in the Col-0, C24, Kas-2, Sha, Hodja, and Cvi-0 accessions are shown. *ΨSRKA*, *ΨSCR1*, and *ARK3^SA^* genes are shown in grey, *ΨSRKC* and *ARK3^SC^* genes are shown in white, and *ΨSB* genes are shown as boxes filled with vertical stripes. Arrows shown inside genetic elements illustrate the 5′ to 3′ orientation of the sequences, and black teeth marks indicate 5′ and 3′ gene truncations. In the Col-0 and C24 *ΨS* haplotypes, the boxes filled with horizontal stripes indicate insertions within the *ΨSCR1* and *ΨSRKA* sequences. In the C24 haplotype, the asterisk marks the deleted *ΨARK3* sequence unique to C24, and the vertical arrow shows the location of the recombination event between *SA* and *SC* haplotypes that produced this haplotype. In the Kas-2 and Sha/Hodja *ΨS* loci, the hatch marks between genes or gene fragments indicate that the distance, orientation, and order of *ARK3* and *ΨS*-locus sequences is not known. Arrows above *ΨSCR1* in Col-0 and the *ΨeSRKA* fragment in C24 indicate the overall orientation of the pseudogenes [27]. The *ΨS*-locus genes are not drawn to scale.

The results of DNA gel blot (Figure 1) and PCR analyses are compiled with previously-reported *S-*locus polymorphism data [27] in Table 2. As depicted in Figure 2, the data reveal three distinct structures for the *ΨS* locus in accessions that exhibit a developmentally-stable transgenic SI phenotype. Consistent with previous results, the Sha and Hodja accessions are inferred to contain very closely related, if not identical, *ΨS* haplotypes that appear to be derived exclusively from an ancestral *SA* haplotype: both accessions lack *ΨeSRKC* and *ΨSRKC* exon 7 sequences and contain the *ARK3^SA^* allele and a truncated *ΨSRKA* sequence containing only *ΨeSRKA* but not *ΨSRKA* exon 7. In contrast, the Kas-2 *ΨS* haplotype, like the C24 *ΨS* haplotype, is clearly an inter-haplotype recombinant: both haplotypes carry the *ARK3^SC^* allele as well as *ΨSRKC* and *ΨeSRKA* sequences. They are not identical, however, as C24 contains only the 3′ portion of *ΨSRKC* and a duplication of *ARK3* sequences [27], while Kas-2 contains a full-length *ΨSRKC* sequence [28] and only one copy of the *ARK3^SC^* allele (Figure 2). Interestingly, C24 was the only accession found to contain the chimeric copy of *ARK3* [27].

**Table 2:** Analysis of *ΨS*-locus haplotypes in selected accessions.

	*ΨSA*			*ΨSC*			*ΨSB*	
Accession	*ΨeSRKA* a	*ΨSRKA* exon 7 a	*ΨSCR1* a	*ΨeSRKC* a	*ΨSRKC* exon 7 a	*ARK3* Indel b	*ΨeSRKB* a	*ΨSCRB* a
Col-0	+	+	+	−	−	−	−	−
Hodja	+	−	−	−	−	−	−	−
Sha	+	−	−	−	−	−	−	−
Kas-2	+	−	−	+	+	+ c	−	−
C24	+	−	−	−	+	+	−	−
Ra-0	−	−	−	+	+	+	−	−
Lz-0	−	−	−	+	+	+	−	−
Mr-0	−	−	−	+	+	+	−	−
Ita-0	−	−	−	+	+	+ c	−	−
Nok-3 d	+	+	+	+	+	+	−	−
Cvi-0	−	−	−	−	−	−	+	+

a DNA gel blot analysis was used to determine whether each accession contains (+) or does not contain (−) sequences corresponding to: the extracellular domain (*eSRK*) and last exon (exon 7) of *ΨSRKA* or *ΨSRKC,* the *ΨSCR1* sequence, the extracellular domain of *ΨSRKB,* and *ΨSCRB*. Results shown in bold type were not reported previously and the corresponding DNA gel blot images are shown in Figure 1. Results shown in regular type were previously reported in [27].

b PCR amplification was used to determine if the *ΨSC*-associated deletion in the first intron of *ARK3* was present (+) or absent (−).

c Polymorphisms in the *ARK3* gene, between exons two and five, rather than the first intron, have been reported [27] and these data support those shown here.

d In addition to the Kas-2 and C24 accessions, this study shows that the *ΨS* locus of the Nok-3 accession also experienced inter-haplotypic recombination.

#### Re-examination of *ΨSCR1* sequences in the Col-0 reference accession

**Figure 3:**
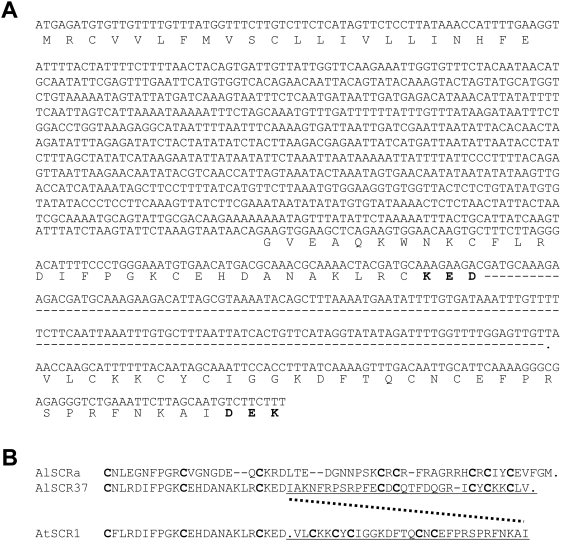
Complete sequence of the *A. thaliana* *Ψ* *SCR1* second exon. (A) DNA and primary amino-acid sequence of the Columbia *ΨSCR1* first exon, intron, and rearranged second exon. The first half of the second exon ends at the “KED” amino acid sequence shown in bold [23]. The insertion after “KED” is marked by the dotted line. The second half of the *ΨSCR1* second exon has been inverted in relation to the rest of *ΨSCR1*. The “DEK” at the end of the sequence is an inverted duplication of the “KED” shown before the insertion. (B) Amino-acid sequence alignment of the second exons of *A. lyrata SCRa*, *AlSCR37,* and *ΨSCR1*. The underlined portions and the dotted line show the sequences that have been inverted in *ΨSCR1* relative to *AlSCR37.* The nucleotide sequence of *AlSCR37* is shown in Figure S1.

*SCR* genes typically contain two small exons, the first encoding the signal peptide and the second encoding the mature ∼50-amino acid long SCR protein. The Col-0 *S* haplotype was previously reported to harbor only a truncated *ΨSCR1* sequence containing exon 1 but lacking approximately half of exon 2 [23]. More recently, exon 1 and part of exon 2 of *A. lyrata SCR37* (*AlSCR37*), the likely ortholog of *ΨSCR1*, were isolated by using primers designed based on the Col-0 *ΨSCR1* sequence [30]. Starting with *A. lyrata S37* plants kindly provided by Drs. Bechsgaard and Schierup (Department of Ecology and Genetics, Institute of Biology, University of Aarhus, Aarhus, Denmark), a PCR approach was employed to clone the missing portion of *AlSCR37* exon 2 using the known *AlSCR37* sequence as an anchor (see Methods). The resulting complete *AlSCR37* sequence (Figure S1; Accession Number FJ752546) was then used to query the Col-0 genome sequence for the missing portion of *ΨSCR1*. As shown in Figure 3, the results of the BLAST search demonstrated that *ΨSCR1* exon 2 is not deleted as previously reported. Rather, the entire exon-2 sequence is present in the Col-0 *S* haplotype, albeit in a highly rearranged configuration: it contains a 142 base-pair insertion and its 3′ portion is inverted and out-of-frame relative to the 5′ segment of the sequence. Because previous surveys of *A. thaliana* accessions had assayed only for the truncated *ΨSCR1* sequence, we surveyed 96 accessions using primers designed to amplify an intact exon-2 sequence lacking the insertion and inversion. However, none of the accessions tested, including C24, Kas-2, Hodja, and Sha, all of which contained remnants of the *SA* haplotype, contained an intact *ΨSCR1* exon 2 (Figure 2). In addition, when querying the accessions shown in Table 2 by DNA gel blot hybridization, only Col-0 and Nok-3 hybridized to the newly-identified portion of *ΨSCR1* exon 2, i.e. the results are the same as those obtained using a probe corresponding to the previously-known *ΨSCR1* sequence [26],[27]. Overall, these data indicate that mutations in the *ΨSCR1* gene arose very early in the evolutionary history of *A. thaliana* accessions containing the *ΨSA* haplotype.

### QTL Analysis of the C24::*AlSRKb-SCRb* x Col-0 Cross

Information on the molecular events associated with the transition from out-crossing to selfing in *A. thaliana* may also be gleaned by genetic analyses of crosses between accessions that differ in expression of SI. In previous studies, genetic analysis of a relatively small C24::*AlSRKb-SCRb* x Col-0 F2 population [26] had inferred the segregation of two loci affecting pollination phenotype and identified a major modifier causing breakdown of SI in close linkage to the Col-0 *ΨS* locus [33].

In this study, we raised a larger F2 population of 300 plants derived by selfing an F1 plant, and we performed a cursory analysis to confirm the hypothesis that two loci with dominance of SI-conferring alleles segregated in this cross. Individual plants were classified into four phenotypic groups based on autonomous seed set: plants producing empty fruits with only an occasional fruit containing seed, similar to the C24::*AlSRKb-SCRb* parent (1 in 80 fruits measured); plants with a full seed set similar to wild-type untransformed plants; plants producing few fruits with seed (1–3 for every 10 fruits measured); and plants producing many fruit with seed (4–8 for every 10 fruits counted). Subsequent manual self-pollination of these plants determined that the number of pollen tubes formed at the stigma surface was consistent with fruit set. Plants in the empty-fruit group exhibited the SI response in all self-pollination assays, while plants with full fruit set exhibited a self-compatible pollination phenotype similar to untransformed plants. Plants that produced few or many fruits with seed exhibited variable pollination phenotypes, in which breakdown of SI occurred in an apparently random fashion in individual flowers, and these plants are classified as being partially self-compatible. In addition, loss of SI was stigma specific as determined by reciprocal pollinations of self-compatible plants with the C24::*AlSRKb-SCRb* parent. The results of a chi-squared test based on the proportions of the phenotypic categories were consistent with segregation of two loci with dominance of SI-conferring alleles (X^2^ = 3.38; p = 0.3). A scan of the genome with molecular markers distributed on all five chromosomes confirmed the presence of a Col-0-derived modifier locus with strong effect located on chromosome 4 near the *ΨS* locus, which corresponds to the previously-identified *S-*locus-linked modifier on chromosome 4 [33]. It also determined that a second Col-0-derived modifier locus responsible for partial self-compatibility was located on the bottom of chromosome 3.

The strong-effect *S*–locus-linked modifier [33] can mask the effects of weak-effect modifiers. Therefore, to ensure detection of weak-effect loci, a QTL mapping population was generated that subtracted the genetic effects of this major modifier (see Methods). This population segregated for self-fertility, as expected. Manual self-pollination of a developing series of stigmas from two representative self-compatible plants revealed weakening of SI and some pollen tube growth in the most mature flowers. In contrast, self-pollination of a developing series of stigmas from two representative self-incompatible plants detected no pollen tubes in mature stigmas. Furthermore, reciprocal pollinations of self-compatible plants with C24::*AlSRKb-SCRb* transformants confirmed that the modifier alleles segregating in this population have stigma-specific effects as in the original C24::*AlSRKb-SCRb* x Col-0 cross. However, the self-compatible trait exhibited low penetrance in this population. On any given self-compatible plant, some flowers would not develop fruits with seeds, due to the SI response, while other flowers would develop into fruits filled with seeds. There was also great variability as to where on the stem SI would break down, the number of flowers that exhibited breakdown of SI, and the strength of the breakdown for each individual flower.

In view of this variability, manual self-pollinations of a small number of randomly-selected individual flowers, as is usually done in pollination assays, cannot reflect overall plant phenotype. Consequently, standard pollination assays are not useful for phenotypic classification of plants in the QTL mapping population. Therefore, we used the size of mature fruit produced by autonomous self-pollination as a measure of the extent of SI breakdown in individual flowers. We reasoned that fruit size was a valid proxy for pollination phenotype because of the known strong correlations between fruit size and number of seeds per fruit (as described previously [34] and confirmed in our study), and between number of seed in a fruit and strength of SI (as observed in our F2 population).

QTL analysis was performed using a total of 186 individuals (see Methods). For phenotypic classification, it was important to distinguish between empty fruits and fruits with few seeds. Based on dissection of 25 of the smallest fruits in this population, it was determined that a mature fruit containing at least one seed had a width of at least 0.6 mm. Therefore, fruits that were narrower than 0.6 mm were classified as being empty and indicative of a self-incompatible response, while fruits that had a width of 0.6 mm or greater were classified as containing seed and indicative of a breakdown of SI. Similar measurements of mature fruit produced by self-incompatible plants in the QTL mapping population gave an average fruit length of 0.42 cm±0.05 (n = 912, with only one fruit in 25 having a width of 0.6 mm), a value very similar to that of the C24::*AlSRKb-SCRb* parental strain, in which average mature fruit length was 0.48 cm±0.07 (n = 80, with only one fruit having a width of 0.6 mm). By comparison, the average length of seed-filled mature fruit in the self-compatible parent of the QTL population was 1.33 cm±0.4 (n = 59), while average fruit lengths in untransformed plants of the C24 and Col-0 accessions were 1.54 cm±0.19 (n = 80) and 1.38 cm±0.07 (n = 80), respectively.

**Figure 4:**
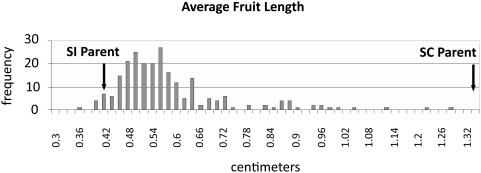
Fruit-length distribution in the QTL mapping population derived from the C24::*AlSRKb-SCRb* x Col-0 cross. A total of 186 individuals were measured for their average fruit length. The normal distribution observed (with a slightly positive skew) is indicative of a multigenic trait, with each gene having an additive effect on the trait value. The average fruit-length values for the self-incompatible (SI) and self-compatible (SC) parents of the mapping population are shown at the lower and upper ends of the distribution, respectively. Fully self-incompatible plants in this population have average fruit-length values equivalent to those of the self-incompatible parent (see text).

**Figure 5:**
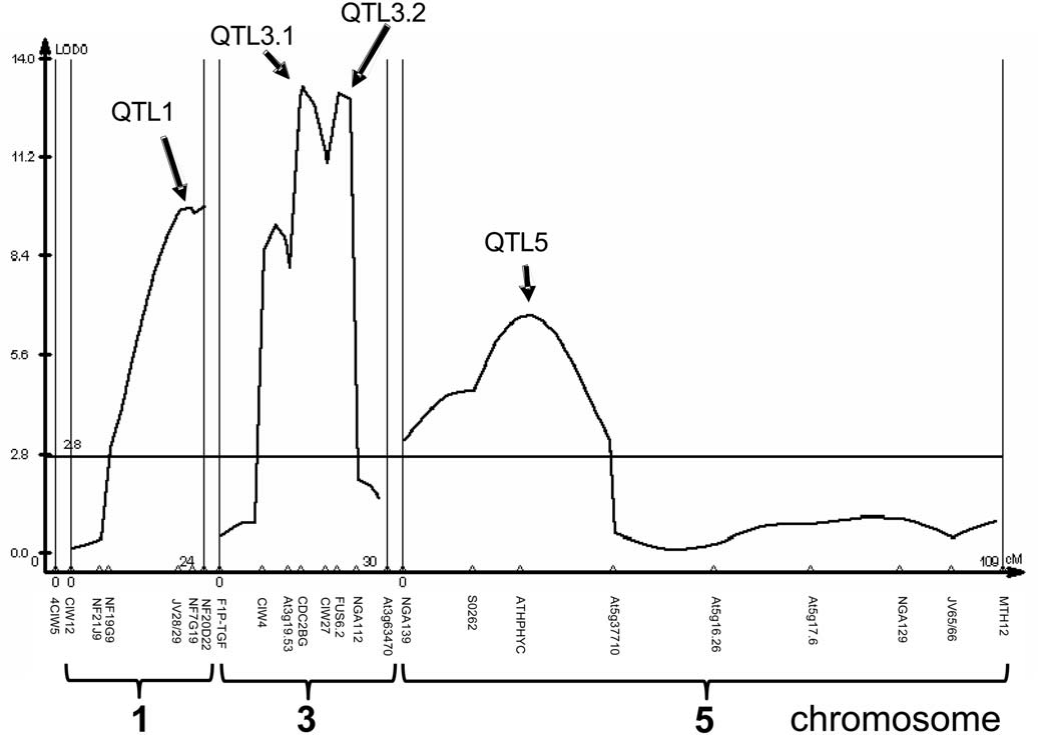
QTL analysis of plants derived from the C24::*AlSRKb-SCRb* X Col-0 cross. The graph shows the QTL identified by their effect on average fruit length. The *x* axis shows the distance between markers in centiMorgans (cM) for each chromosome and the identity and relative position of these markers are shown below the *x* axis. The *y* axis is shown as a LOD (logarithm of odds) score for each position on the *x* axis. Although all QTL were found to fall well above the empirically-determined significance threshold (shown by the horizontal line), only QTL3.2 on chromosome 3 has been directly associated with breakdown of SI in this population. The peak to the left of QTL3.1 was not classified as a QTL because the “trough” separating the two peaks was not sufficiently deep (2-LOD interval).

As shown in Figure 4, the trait value distribution for the mapping population was continuous and approximately normal, suggesting the involvement of several genes in the control of fruit length. Individual plants were genotyped using 24 markers, microsatellites, and single nucleotide polymorphisms in chromosomal regions that segregated for Col-0-derived sequences. As shown in Figure 5 and Table 3, four QTL underlying the observed differences in fruit length were found: two QTL (QTL3.1 and QTL3.2) on chromosome 3, one QTL (QTL5) on chromosome 5, and one QTL (QTL1) on chromosome 1, which accounted respectively for 25%, 24%, 15%, and 16%, of the observed variation in fruit length. All of the QTL regions were well above the significance threshold, and none corresponded to “minor QTL” with peaks near the threshold line.

**Table 3:** QTL analysis of the C24::*AlSRKb-SCRb* x Col-0 cross.

	QTL1	QTL3.1	QTL3.2	QTL5
Nearest Marker	NF20D22	CDC2BG	FUS6.2	ATHPHYC
NIL Introgression (Mbp) a	12.5	10	1	20
Breakdown of SI in NIL	no	no	yes	no
Max LOD Score b	9.75	13.22	13.08	6.75
2 LOD Interval (cM)	8.6	5.4	4.7	20.7
% of Total Variance	16	25	24	15

a The NIL for QTL3.1 encompasses the small peak to the left of QTL3.1 shown in Figure 5. All NILs were tested for breakdown of SI by examining manually self-pollinated flowers over the course of floral development and by observation of seed set. NILs corresponding to QTL1, QTL3.1, and QTL5 exhibited no seed set and were SI at all developmental stages. NIL3.2, corresponding to QTL3.2, set noticeably more seed than the other NILs, though less than Col-0::*AlSRKb-SCRb*. A total of 29 developmental series of four consecutive manually self-pollinated flowers were used to assess NIL3.2. Only in three of those series was a weak breakdown of SI observed, and only in mature flowers.

b The Max LOD Score indicates the likelihood score directly under a QTL peak.

Nearly isogenic lines (NIL) were generated for each QTL region. Among these, only one NIL exhibited a breakdown of SI, as determined by manual self-pollination of flowers over the course of stigma development and by observation of autonomous seed set (Table 3). This NIL, NIL3.2, incorporates QTL3.2 and likely corresponds to the chromosome-3 modifier that was associated with partial self-compatibility in the original C24::*AlSRKb-SCRb* x Col-0 F2 population. Epistasis between QTL1, QTL3.1, and QTL5 was assessed by crossing the corresponding NILs to generate “double NILs”. However, none of the “double NILs” showed a breakdown of SI based on observations of seed set. A possible explanation for this result is that these QTL do contribute to breakdown of SI, but their effect may only be detected when all three are combined with QTL3.2. Another possibility is that QTL1, QTL3.1, and QTL5 control SI-independent variation in fruit length. Although the SI response exerts the major influence on seed number and consequently on fruit size in the populations we analyzed, modifier loci affecting differences in fruit size and seed number per fruit between wild-type Col-0 and C24 may also be segregating, similar to the loci uncovered in a previous analysis of natural variation for various fruit parameters [34]. Interestingly, this earlier study of fruit length differences between the Cvi and Landsberg accessions had identified a QTL in the QTL3.1 region [34], but not in the other QTL regions identified in this study.

In an attempt to fine-map QTL3.2, an F2 mapping population was generated by crossing an NIL3.2 plant with a wild type (untransformed) C24 plant. This population segregated for the 1-megabase Col-0 introgression encompassing QTL3.2 (Table 3). F2 plants exhibiting recombination within the QTL3.2 region were identified by screening 2,016 individual plants, both phenotypically for seed set and genotypically with markers “NGA12” and “intron2” located just inside the introgressed region (Table S1). Three phenotypic groups were observed among recombinant plants: self-incompatible, self-compatible, and surprisingly, partially self-compatible. The occurrence of partially self-compatible plants in the recombinant pool was not expected because the gene underlying QTL3.2 was determined to be recessive in the Col-0 background. Also unexpectedly, these recombinants did not show a tight correlation between genotype and phenotype under the assumption of complete dominance of the SI-conferring C24 allele (Table S2). Nevertheless, F3 families were generated from self-compatible NIL3.2 F2 plants. Analysis of nine such NIL3.2 F3 families failed to identify self-compatible plants in six of those families, indicating that the self-compatibility phenotype can be completely erased from one generation to the next (Table S2). In view of this result, the genotype-to-phenotype correlations inferred for the self-incompatible class of NIL3.2 F2 plants become questionable. Nevertheless, with this caveat in mind and considering only the unambiguous self-compatible NIL3.2 F2 plants, QTL3.2 is tentatively mapped to a region of approximately 105,000 base pairs between genes At3g60440 and At3g60730 (Table S2 and Table S3).

## Discussion

Our results have extended our understanding of the genetic events at the *S* locus and at modifier loci that accompanied the switch to self-fertility in *A. thaliana.*

The identification of four accessions, in addition to C24, in which self-fertility may be clearly attributed to a non-functional *S* locus is significant for several reasons. From a practical point of view, the availability of several strains with diverged genetic backgrounds that do not contribute SI modifier alleles in crosses to laboratory-generated mutants will greatly facilitate the mapping of these mutants and the eventual cloning of genes required for SI. From an evolutionary perspective, the finding demonstrates that rather than being unique, the C24 accession is only one of potentially many accessions whose self-fertile phenotype may be fully reverted to SI by transformation with the *AlSRKb-SCRb* genes. Interestingly, these accessions are not confined to one geographical region: C24 is a southern-European accession originally isolated in Portugal, whereas Kas-2, Hodja, and Sha are all central Asian accessions from Kashmir (Kas-2) or Tajikistan (Hodja and Sha), and Cvi-0 is restricted to the Cape Verdi Islands. A genome-wide polymorphism study in which 876 loci spread across the genome were surveyed in 96 accessions [35] had indicated that all accessions isolated from Tajikistan are genetically very similar to one another (although Hodja was not included in the study), that Sha and Kas-2 are very closely related to each other, and that both are significantly diverged from C24 and Cvi-0, which in turn are also highly diverged from each other.

**Figure 6:**
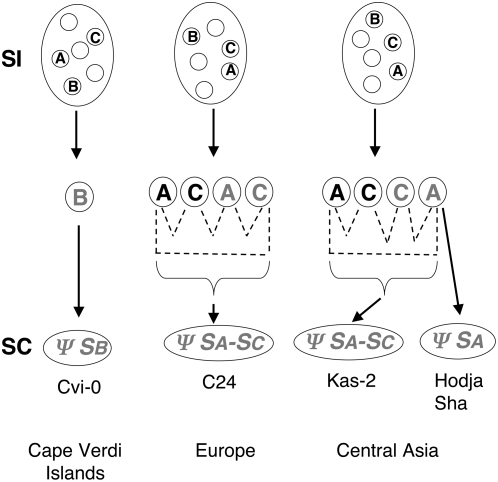
Proposed paths for generation of extant *ΨS* haplotypes from ancestral *S* haplotypes in different geographical locations. In the diagram, functional and non-functional *ΨS* haplotypes *S* haplotypes are depicted in black and grey letters, respectively. Ovals in the top row represent ancestral self-incompatible (SI) populations harboring multiple *S* haplotypes (small circles), including *SA, SB,* and *SC* (A, B, and C). The *SA, SB,* and *SC* haplotypes are shown to have undergone independent inactivation in different geographical locations. In the Cape Verdi islands, this process would have produced *ΨSB.* In southwestern Europe and central Asia, *SA-SC* inter-haplotypic recombination events (dashed lines) would have occurred in *S-*locus heterozygotes, either between functional haplotypes (generating a non-functional recombinant haplotype) or after inactivation of one or both *S* haplotypes, to produce the C24 and Kas-2 *ΨSA-SC* recombinant haplotypes. The double-headed arrow reflects the uncertain relationship between the Kas-2 *ΨSA-SC* and the Hodja/Sha *ΨSA* haplotypes: the latter might have arisen independently by decay of an ancestral *SA* haplotype or it might have been derived from a Kas-2-like *ΨSA-SC* haplotype by loss of *SC* sequences.

Our analysis of the C24, Cvi-0, Kas-2, Hodja, and Sha accessions has illuminated the genetic events that likely caused loss of SI in these accessions and potentially others with similar *ΨS*-loci, genome-wide polymorphisms, and provenance. Keeping in mind that the *ΨSA, ΨSB,* and *ΨSC* haplotypes were derived from distinct ancestral functional *S* haplotypes, the four haplotypic structures observed in C24, Cvi-0, Kas-2, and the Hodja/Sha group (Figure 2) are consistent with independent origins of these *ΨS* haplotypes. The Cvi-0 *ΨSB* haplotype, which lacks *ΨSA* and *ΨSC* sequences was clearly independently derived. The Sha and Hodja *ΨS* haplotypes are highly-decayed versions of the ancestral *SA* haplotype also found in Col-0, and it is possible that the *S* haplotypes in these three accessions might have been derived from the same *ΨSA* haplotype. In contrast, the C24 and Kas-2 *ΨS* haplotypes are both recombinant haplotypes generated by illegitimate recombination between ancestral *SA* and *SC* haplotypes. It is possible that the C24 *ΨS* haplotype was derived from a Kas-2-like *ΨS* haplotype via a complex series of restructuring events. Alternatively, based on the extensive genome-wide divergence inferred for the C24 and Kas-2 accessions [35], their recombinant *ΨS* haplotypes might have arisen independently, as illustrated in Figure 6.

Our data thus demonstrate that the ability to express a developmentally-stable transgenic SI response is not restricted to one group of highly-related accessions or to accessions harboring one *ΨS* haplotype. Additionally, the divergence of *ΨS* haplotypes harbored by these accessions provides further evidence for the lack of a single selective sweep at the *A. thaliana ΨS* locus [27],[29]. Rather, the results support the hypothesis that the switch to self-fertility in this species occurred by recurrent selection of distinct *S-*locus loss-of-function mutations. Such a process involving selection of adaptive mutations of independent origins has been referred to as a “soft sweep” [36]. Notably, soft sweeps are not restricted to the switch to self-fertility described here, and evidence of their occurrence is suggested by studies of polymorphisms in a variety of systems and organisms ranging from protozoa to human [36]. For example, in three-spine stickleback fish, selection for reduced body-plate armor in isolated European and Japanese populations has apparently resulted in the fixation of different alleles of ectodysplasin, a factor required for epithelial cell morphogenesis [37],[38].

**Figure 7:**
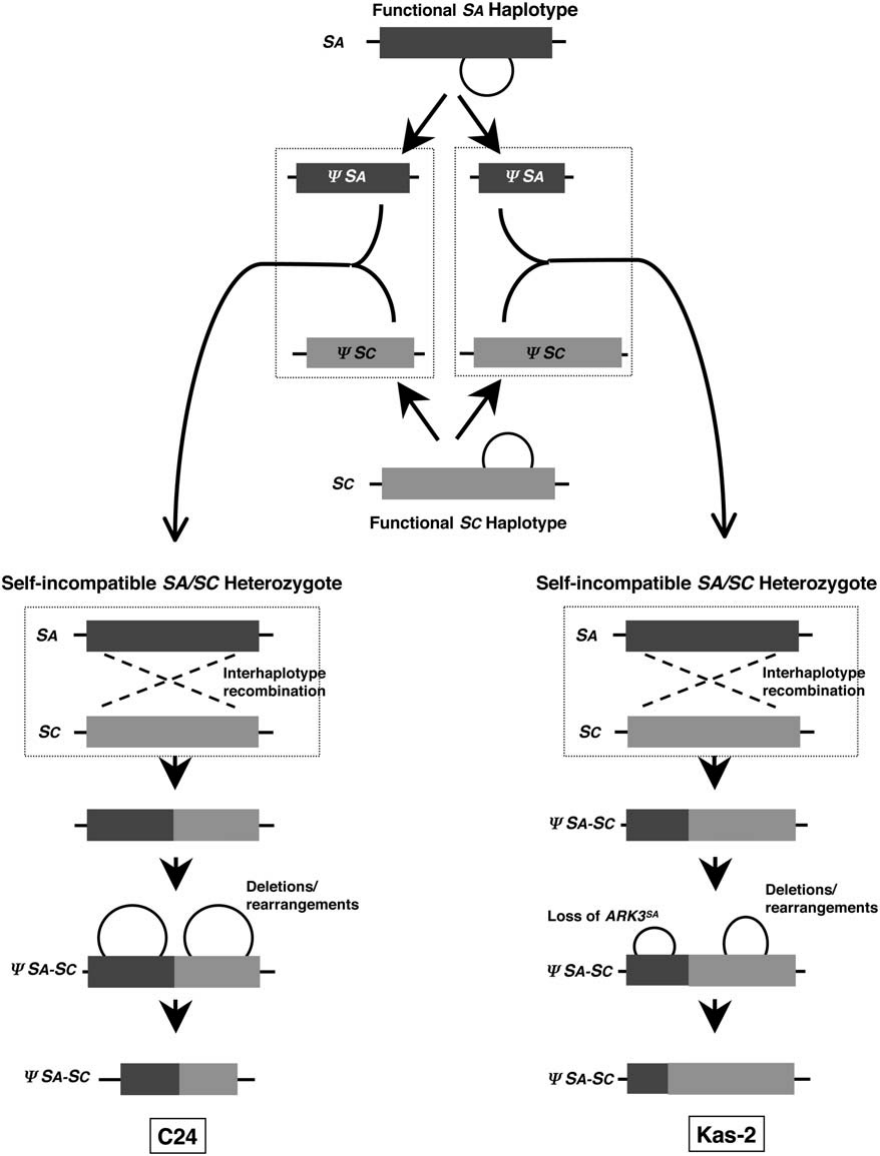
Scenarios for the independent origin of the C24 and Kas-2 *ΨSA-SC* recombinant haplotypes. The diagram illustrates how the C24 and Kas-2 *ΨS* haplotypes might have been generated by independent events occurring in distinct individuals. The individuals in which the postulated recombination events occurred are framed by dashed boxes. Deletions and rearrangements are shown by circles. Two possibilities are shown. (1) In the left and right diagrams, distinct crossover events between *SA* and *SC* haplotypes occur in different self-incompatible heterozygous individuals causing *S-*locus inactivation; subsequent restructuring by deletions and rearrangements generates the C24 (left) and the Kas-2 *ΨSA-SC* haplotypes (right). (2) In the center diagram, *SA* and *SC* haplotypes are inactivated by distinct restructuring events to generate different versions of *ΨSA* and *ΨSC* haplotypes. Subsequent crossover events in self-fertile heterozygous individuals carrying different combinations of these non-functional haplotypes then generate the C24 and Kas-2 *ΨSA-SC* haplotypes.

**Figure 8:**
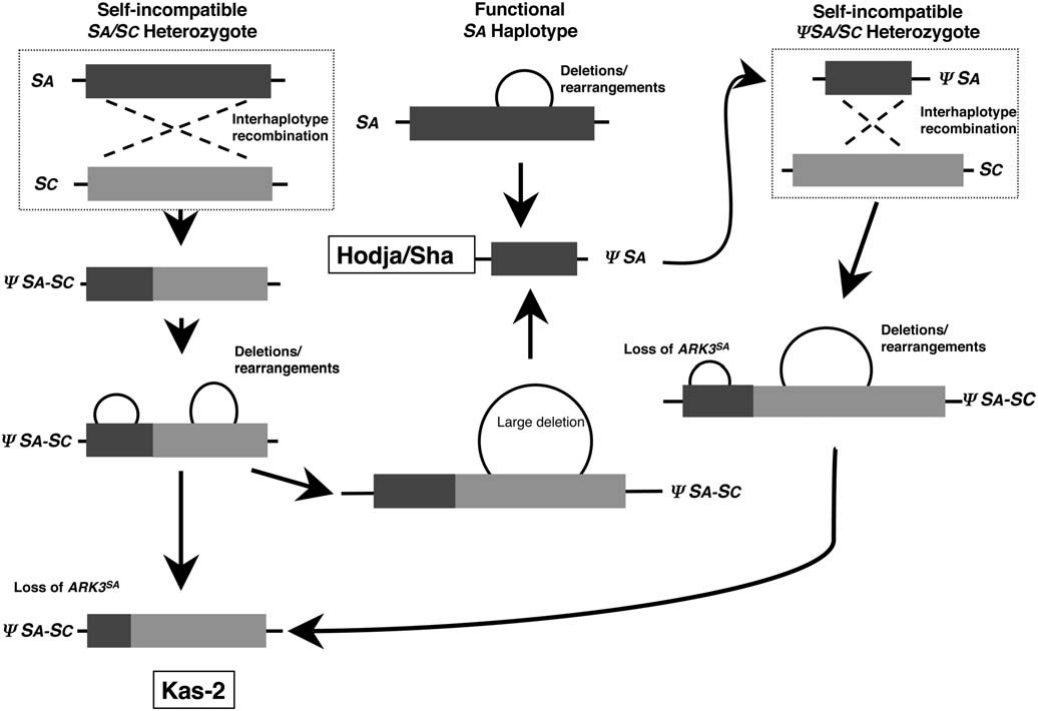
Scenarios for the origin of the Kas-2 and Hodja/Sha *ΨS* haplotypes. The diagram illustrates how the Hodja/Sha and Kas-2 *ΨS* haplotypes might have been generated by independent events occurring in distinct individuals and how these haplotypes might have been produced from shared *ΨS-*locus intermediate configurations. The individuals in which the postulated recombination events occurred are framed by dashed boxes. Deletions and rearrangements are shown by circles. To the left, the Kas-2 *ΨSA-SC* is generated as in Figure 7. In the center, the Hodja/Sha *ΨSA* haplotype is generated either from deletions and rearrangements occurring in a functional *SA* haplotype (top) or from a progenitor of the Kas-2 *ΨSA-SC* haplotype (bottom). To the right, an alternative path for the generation of the Kas-2 *ΨSA-SC* haplotype involves a crossover event between a Hodja/Sha-like *ΨSA* haplotype and a functional *SC* haplotype.

Possible scenarios for the generation of the observed *ΨS* haplotypes are shown in Figures 7 and 8. It should be noted however, that the exact nature of the inactivating mutation and sequence of events that produced these *ΨS* haplotypes cannot be inferred from our data. A major difficulty in charting the history of the *A. thaliana S* locus is distinguishing a primary inactivating mutation from subsequent decay of the non-functional haplotype by further mutation, sequence loss, and rearrangement. For example, it is impossible to know whether the recombination events that produced the C24 and Kas-2 *S* haplotypes caused *S-*locus inactivation by disrupting the physical linkage between functional allelic *SRK-SCR* pairs, or if they occurred between already-mutated *SA* and/or *SC* haplotypes. There is also uncertainty as to whether the Kas-2 primary mutation is the same as that of Hodja and Sha. Although all three accessions have closely-related genomes and originate from close geographical locations, their *ΨS* loci differ in allele content and extent of decay. Furthermore, in contrast to the *ΨSA* haplotypes and the *ΨSB* haplotype of Cvi-0, for which both *ΨSRK* and *ΨSCR* sequences as well as their *A. lyrata* orthologues are known, only an incomplete picture of *ΨSC* haplotypes is available because neither *A. thaliana ΨSCRC* sequences nor the orthologous *A. lyrata SCR36* (*AlSCR36*) sequences have as yet been isolated. Identification of *AlSCR36* is likely to be particularly informative. Just as *AlSCR37* sequences allowed a resolution of the Col-0 *ΨSCR1* structure in this study, *AlSCR36* sequences may be used to investigate the fate of the *SCRC* allele in *A. thaliana* and to determine if, and in what form, these sequences were maintained in Kas-2, C24, or other *ΨSRKC*-carrying accessions.

The structures of the *ΨS* haplotypes observed for Kas-2 and C24 as well as Nok-3 (Table 2) reveal an important role for recombination in shaping extant *S-*locus structure in *A. thaliana.* The *ΨSA-ΨSC* recombinant haplotypes of these accessions provide clear evidence for the occurrence of inter-haplotype recombination events in geographical areas where the *SA* and *SC* haplotypes were both present [27], as in southwestern Europe for the C24 *ΨS* haplotype and in central Asia for the Kas-2 *ΨS* haplotype (Figure 6). Only the *ΨSB* haplotype, which is restricted to the Cape Verdi Islands, did not participate in inter-haplotype recombination (Figure 6). Thus, recombination between *S* haplotypes that encode different SI specificities can occur, despite the extensive structural heteromorphism and sequence divergence that typically distinguish these *S* haplotypes. It is possible that DNA crossover might occur in small regions of sequence similarity, such as regions containing the many transposon-like sequences present within the locus [27].

The contrast between the occurrence of inter-haplotype recombination events inferred in this study and the very low effective rate of recombination that typically characterizes the *S-*locus region in self-incompatible species [15],[17] suggests that purifying selection against recombinants actively maintains low rates of recombination in the region, as previously discussed [17]. The switch to self-fertility is expected to have caused relaxation of this selective pressure, leading to further restructuring of the *S*-locus region. Thus, it is interesting to consider whether current recombination rates at the *ΨS* locus of *A. thaliana* are consistent with this expectation. The potential for recombination certainly exists despite high levels of self-fertility, as gene flow via pollen dissemination has been shown to contribute to genetic variability in local populations of the species [39]. Furthermore, the *S*-locus region was identified as a recombination hotspot in a cross between the Col-0 and Ler-0 accessions [40]. However, these two accessions harbor highly similar if not identical *ΨSA* haplotypes [27], and much lower recombination rates are expected in crosses involving structurally-divergent *ΨS* haplotypes. This expectation was confirmed by a recent analysis of 3,210 plants derived from a cross between C24 and RLD, an accession that carries the same *ΨSA* haplotype as Col-0 (Figure 2). Using the *S-*locus flanking markers *PUB8* (At4g21350) and *ARK3* (At4g21380), which are separated by 34 kilobases in RLD, only 1 recombinant was recovered, and this recombinant was produced by a cross-over event within the promoter region of *PUB8,* not within the *S* locus proper [33]. Thus, the likelihood of further *S-*locus restructuring by recombination between structurally-diverged *ΨS* haplotypes is low, despite relaxed selection on the locus.

The acquisition of a robust and developmentally-stable SI response by accessions that harbor independently-derived *ΨS* haplotypes provides the strongest evidence to date that *A. thaliana* evolved from an obligate out-crosser to a predominantly selfing species through multiple *S*-locus inactivating mutations in distinct outbreeding individuals. One interpretation of our data is that self-fertility in *A. thaliana* arose at least twice: once in an *SA* or *SC* haplotype (producing the Hodja/Sha, C24, and Kas-2 *ΨS* haplotypes) and once in an *SB* haplotype (producing the Cvi-0 *ΨSB* haplotype). A less conservative interpretation would invoke three origins of self-fertility if the C24 and Kas-2 *S* haplotypes are assumed to have arisen independently (Figure 6).

When and how frequently mutations at SI modifier loci occurred in *A. thaliana* must await the molecular cloning of these loci. At least one such SI modifier was uncovered in our QTL analysis of differences in expression of SI between *AlSRKb-SCRb* transformants of the C24 and Col-0 accessions. This previously-unidentified recessive modifier, defined by QTL3.2, was associated with self-fertility in Col-0 and was mapped to chromosome 3. However, phenotypic instability, low heritability, and erasure of the self-compatibility trait in advanced mapping populations precluded further fine mapping and isolation of the underlying gene(s). The cause of this instability is not known. One intriguing possibility is that it might reflect an epigenetic component in control of the self-compatibility trait in these populations. Indeed, phenotypic instability is a hallmark of epigenetically-controlled traits in various organisms [41]–[44]. Furthermore, examples of naturally-occurring epialleles have been reported in plants [43],[45], and widespread epigenetic natural variation has been noted among accessions of *A. thaliana* [46]–[48]. Similar to other epialleles that display unpredictable patterns of instability, the instability of QTL3.2 might be due to the loss of an unlinked *trans-*acting “maintainer” locus through segregation in NIL populations.

In any case, our identification of an unstable modifier of SI has relevance for theoretical modeling and mechanistic studies of switches to self-fertility in *A. thaliana* and other plant species. Clearly, approaches more suited to the identification of unstable alleles than traditional QTL analysis and association mapping [49] will be required to clone at least some of the genes associated with self-fertility. Future molecular genetic analysis of polymorphisms at SI modifier loci, as well as investigation of *S-*locus structure in additional accessions that might express developmentally-stable SI upon transformation with the *AlSRKb-SCRb* genes, will undoubtedly determine if switches to self-fertility occurred exclusively by inactivation of the *S* locus in the *A. thaliana* lineage.

## Methods

### Plant Growth Conditions and Transformations

*A. thaliana* plants were typically grown at 22°C and a photoperiod of 16 hours. Plants that were used for transformation by the floral dip method [50] were grown under a 24-hour photoperiod. All accessions used in this study were obtained from the Arabidopsis Biological Resource Center in Columbus, Ohio. The Kashmir (Kas-2; CS22638), Shahkdara (Sha; CS929), and Hodja-Obi-Garm (Hodja; CS6178) accessions were transformed with the p548 plasmid (here designated *AlSRKb-SCRb*), a previously-described pBIN-PLUS derivative containing the *A. lyrata SRKb* and *SCRb* genes [26]. DNA gel blot analysis was used to confirm the independent origin of transformants and to identify transformed lines carrying single integrations of the transgene pair: genomic DNA was isolated from individual plants by the CTAB method [51], digested with EcoR1, transferred to Hybond H+ membrane (Amersham Biosciences, Piscataway, NJ), and hybridized according to the Hybond H+ membrane instruction manual with a probe specific for the *Neomycin PhosphoTransferase (NPTII*) gene that was labeled with ^32^P using the Random Priming kit (Roche, Indianapolis, IN). Hybridized membranes were washed at 65°C first in a solution containing 2× SSC and 0.5% SDS and subsequently in a solution containing 0.2× SSC and 0.5% SDS. Blots were exposed to phosphor screens, scanned using a GE Healthcare STORM phosphorimager (Piscataway, NJ), and analyzed with the ImageQuant software package purchased as a bundle with the phosphorimager. In all cases analyzed, each transformant was found to exhibit a unique transgene pattern (data not shown), consistent with independent transgene integration events and demonstrating that each of the analyzed transformants was or independent origin.

### Pollination Assays

Pollination responses were tested on pollen-free stigmas just before anthesis, when the stigmas are receptive to pollen but before the pollen grains are mature and released from the anthers. Using a stereomicroscope, stigmas were manually pollinated with hundreds of pollen grains from the dehisced anthers of mature flowers. Two hours after pollination, flowers were fixed for 10 minutes in a 3∶1 mixture of ethanol and acetic acid at 65°C, softened for 10 minutes in 1N NaOH at 65°C, washed two times in water, stained in decolorized aniline blue, and transferred to a slide for observation by epifluorescence microscopy [52]. Under these conditions, a pollination is scored as strongly incompatible if no or fewer than 5 pollen tubes are observed per pollinated stigma, as fully compatible when more than 50 pollen tubes are observed per pollinated stigma, and as partially self-compatible (or weakly self-incompatible) when intermediate numbers of pollen tubes are observed.

### Analysis of *ΨS* Loci in Various Accessions

Genomic DNA gel blot analysis with probes derived from different *ΨSRKs* was used to assess the composition of the *S* locus in various accessions of *A. thaliana*. This method is more suitable than amplification by the polymerase chain reaction (PCR) for our study because of the known or expected sequence divergence of the loci under study. Indeed, previous applications of this method to analysis of *S-*locus polymorphisms in *A. thaliana* have demonstrated that it can identify homologous sequences that are missed by PCR (27). Under low-stringency hybridization and washing conditions, DNA gel blot analysis can detect sequences that share as little as 50% overall similarity with the probe but not small stretches of sequence similarity or sequences that have decayed to below the 50% sequence similarity threshold. The probes for this analysis were fragments corresponding to the first exon and the seventh or last exon of *A. thaliana ΨSRKA* (At4g21370) from Columbia (Col-0; CS1092), to the first intron of *ΨSRKB* and *ΨSCRB* from the Cape Verdi Islands accession (Cvi-0; CS1096), and to the first intron of *ΨSRKC* from the Ibel Tazekka accession (Ita-0; CS1244). Fragments were amplified from genomic DNA using specific primers (Table S1), labeled with ^32^P, and used in sequential hybridizations of EcoRI-digested genomic DNA isolated from various accessions, as described above. An insertion/deletion polymorphism in *ARK3* [27], a gene tightly linked to the *S* locus in *Arabidopsis* species, was also assessed by PCR using specific primers (Table S1) to distinguish between the *ARK3^SC^* allele (characteristic of *ΨSC* haplotypes), which has the deletion, and the *ARK3^SA^* allele (characteristic of *ΨSA* haplotypes), which lacks the deletion. Accessions used in this analysis included Kashmir (Kas-2; CS1264), Shahkdara (Sha; CS929), Hodja-Obi-Garm (Hodja; CS6178), C24 (CS906), Col-0, Lezoux (Lz-0; CS22615), Noordwijk (Nok-3; CS22643), Randan (Ra-0; CS22632), Ita-0, Monte (Mr-0; CS22640), and Cape Verdi Islands (Cvi-0; CS902 and CS1096). Standard PCR reagents were used with 35 cycles of the following: 94°C for 30 seconds, 55°C for 30 seconds, and 72°C for one minute or longer.

The accessions were also assayed for previously-unidentified *AtΨSCR1* exon 2 sequences, which were isolated in this study as follows. A recently-reported partial sequence of the *A. lyrata SCR37* (*AlSCR37*) gene, the ortholog of *A. thaliana ΨSCR1* in Col-0 [30], was used as anchor to clone the remainder of *AlSCR37* using the “DNA Walking SpeedUp Premix Kit II” (Seegene, Rockville, MD) and gene-specific primers (Table S1). Amplification of *AlSCR37* genomic DNA (kindly provided by Dr. Jesper Bechsgaard) was performed according to the manufacturer's directions and amplified products were cloned into pGemT-easy (Promega, Madison, WI). Inserts were sequenced at the Cornell University Life Sciences Core Laboratories Center (Ithaca, NY) using SP6 and T7 universal primers. A BLAST search of the *A. thaliana* Col-0 genome using the newly-identified *A. lyrata SCR37* second exon located the corresponding portion of *A. thaliana ΨSCR1*, and primers were designed (Table S1) to screen for the presence of an intact *AtΨSCR1* second exon in 96 accessions of *A. thaliana* [35] using *A. lyrata S37* DNA as positive control.

### QTL Analysis of Col-0 SI Modifiers

SI prevents self pollen from reaching and fertilizing the ovule, and thus precludes fruit expansion. A breakdown or absence of SI allows self pollen to fertilize the ovules, resulting in fruit expansion and elongation. Consequently, for QTL analysis, fruit size was used as a proxy for self-pollination phenotype. Data used to calculate the phenotype values for individual plants were collected by sampling three inflorescence stems, scanning them using a flat-bed scanner, and measuring the length and width of each fruit using ImageJ software (http://rsb.info.nih.gov/ij/). An average of 80 fruits were scanned and measured for each plant, and on average across the population, one-fourth of those fruits contained seeds and were used in the average length calculation. Each of these fruits was the result of autonomous self-pollination, because they were grown in the absence of pollinators. A flower was deemed self-compatible, if the fruit width was greater than 0.6 mm, i.e. the minimal width of one fully-developed seed. Because of variability in fruit development, the trait values reported here were calculated for each plant as the average length of fruits with at least one seed.

The QTL mapping population was generated using a self-fertile F4 plant derived from the C24::*AlSRKb-SCRb* x Col-0 cross, which was homozygous for the *PUB8^C24^* allele and for the Col-0 allele at the chromosome-3 modifier. The F2 parent of the selected F4 plant displayed a transient SI phenotype as determined by seed set and pollination assays (<5 pollen tubes/stigma in young buds and >50 pollen tubes/stigma in older buds and flowers). The F4 plant also produced abundant seed, although some flowers remained self-incompatible throughout development and did not produce seeds. It was homozygous over most of its genome, with Col-0-derived DNA occurring in large stretches on chromosomes 1, 3, and 5, and in a small region on chromosome 4. This plant was back-crossed to C24, producing F4BC progenies that were self-incompatible, similar to the original C24::*AlSRKb-SCRb* x Col-0 F1 hybrid. The F4BC was subjected to forced selfing in immature floral buds (i.e. before stigmas acquire the ability to reject self pollen) to generate an F4BCF2 population for QTL analysis, which we refer to as the QTL mapping population.

Since the C24 accession was not completely sequenced when this study was undertaken, a search for markers that showed co-dominant polymorphisms between C24 and Col-0 was done by PCR screening of publicly available microsattelite markers designed for other pairs of accessions and of random amplification of repetitive elements found in the Col-0 genome (www.arabidopsis.org). In addition, a limited number of dominant SNP markers were designed to detect differences as small as one base pair between the two parents. Twenty-four marker loci (Table S1) were found to be polymorphic between the two accessions and were scored on 186 individuals in the QTL mapping population. Markers were amplified using forward primers with M13 adapters to enable large scale genotyping [53]. A linkage map and mapping files containing genotype and phenotype data were produced using MapManager for analysis in MapManager and also exported into WinQTL Cartographer (http://statgen.ncsu.edu/qtlcart/). All recombination distances, measured in centiMorgans (cM), were co-linear with physical distances (data not shown). QTL interval mapping and composite interval mapping methods were applied to the genotype and marker data using both software programs. The various analyses and programs all produced similar results. A 0.05 significance threshold of LOD 2.8 was determined in WinQTL (http://statgen.ncsu.edu/qtlcart/) by creating a random distribution of the data through 1000 permutations.

## Supporting Information

Figure S1 Sequence of *A. lyrata SCR37*.
(0.09 MB DOC)

Table S1 Primers used in this study.
(0.07 MB DOC)

Table S2 Fine mapping using NIL3.2 plants.
(0.04 MB DOC)

Table S3 Pollination phenotypes in two NIL3.2 F3 families that segregated for self-compatibility.
(0.04 MB DOC)
