# Supplementary material for: Independent S-Locus Mutations Caused Self-Fertility in Arabidopsis thaliana
Source: PLoS Genet. 2009 Mar 20;5(3):e1000426. doi: 10.1371/journal.pgen.1000426 (PMC2650789; doi:10.1371/journal.pgen.1000426)

**Figure S1. Sequence of *A. lyrata SCR37.*** The missing portion of *A. lyrata SCR37* was amplified (see Methods) and cloned into the pGemT-easy plasmid. The gene sequence, including intron and 3’ sequences, is shown along with the predicted amino-acid sequence. The amino-acid sequence shown in bold corresponds to the sequence that was determined in this study. The underlined sequence corresponds to the “TSP3” primer listed in Table S1.


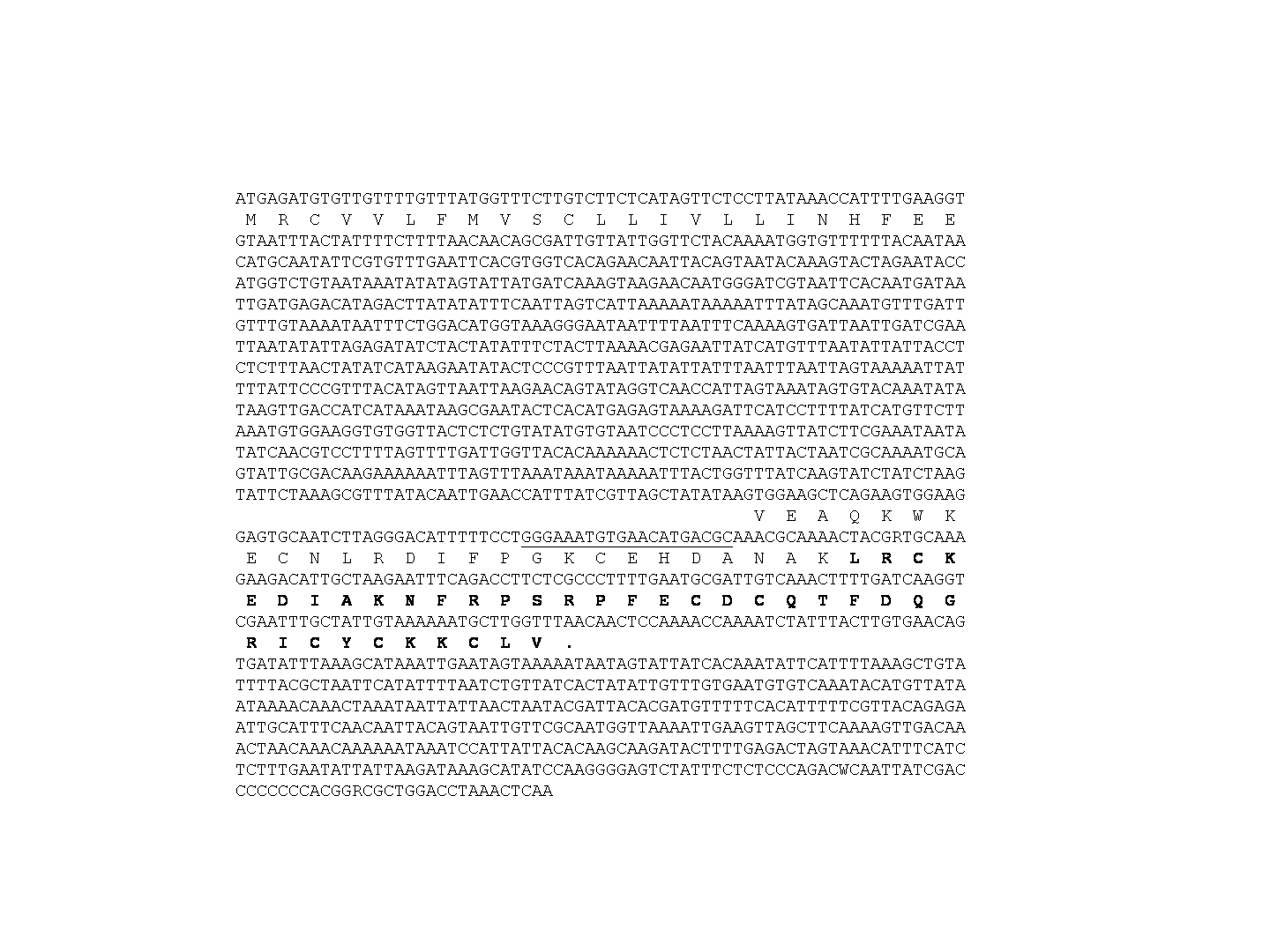

Supplement: Figure S1 — Sequence of A. lyrata SCR37. (0.09 MB DOC) [file pgen.1000426.s001.doc]
