# Supplementary material for: Independent S-Locus Mutations Caused Self-Fertility in Arabidopsis thaliana
Source: PLoS Genet. 2009 Mar 20;5(3):e1000426. doi: 10.1371/journal.pgen.1000426 (PMC2650789; doi:10.1371/journal.pgen.1000426)
Supplement: Table S1 — Primers used in this study. (0.07 MB DOC) [file pgen.1000426.s002.doc]

**Table S1. Primers used in this study.**

**A. Primers used for analysis of** *Ψ****S-*locus structure**

| Primer Name | **Sequence Amplified** | **Primer Sequence** |
| --- | --- | --- |
|  |  |  |
| TSP1fp | A. lyrata SCR37 | GCTATATAAGTGGAAGCTCAGAAGTGGAAGGAG |
| TSP2.fp | A. lyrata SCR37 | GGAAGGAGTGCAATCTTAGGGACATTTTTCC |
| TSP3fp | A. lyrata SCR37 | GGGAAATGTGAACATGACGCAAACGC |
| NB.SCR37.exon1.FP | *AtΨSCR1* | ggtttcttgtcttctcatagttctcc |
| SCR37.KpnI.RP | *AtΨSCR1* | GGCggtaccTTAAACCAAGCATTTTTTACAATAGC |
| AlHapA.KpnI.fp | Col-0 *eSRKA* | GGCggtaccATGAGAGGTGCAGTACCAAAC |
| AtHapA.Col.SacI.rp | Col-0 *eSRKA* | GACATgagctcTCCGATCCATATTATACAACCTG |
| *Ψ*SRKAcol3.fp | Col-0 *ΨSRKA* exon 7 | TCGGTGGTTTTAATGCTCGGTAGCG |
| *Ψ*SRKAcol3.rp | Col-0 *ΨSRKA* exon 7 | GTCCCGTTCTGTACTCGATGACGAATC |
| ARK3colPoly.FP | Col-0 *ARK3* | CTTGATTGAGATGTTGACGCATTCGGTC |
| ARK3colPoly.RP | Col-0 *ARK3* | CGCTCACTCCAATACTCGAACCTATG |
| AtHapC.Kas.KpnI.fp | Ita *eSRKC* | GGCggtaccATGAAAGGTGTACGAAAACCCTAC |
| AtHapC.Kas.SacI.rp | Ita *eSRKC* | GACATgagctcACCAGTCCAAATCACACAAC |
| *Ψ*SRKCkas3.fp | Ita *ΨSRKC* exon 7 | ACCACAACCATTCCTCAACCTAAGAC |
| *Ψ*SRKCkas3.rp | Ita *ΨSRKC* exon 7 | TTACCGAGCTTCAAGAACAGAGACAGTTATTTCG |
| 3’ARK3c24.fp | C24 *ΔARK3* | GCCTCGACTCTCATCTATTTGGTTAGTGTCTC |
| 3’ARK3c24.rp | C24 *ΔARK3* | GCTGTTTACTCGACGAAGAATCAGTGTCAAG |
|  |  |  |

**B. Primers used for QTL analysis**

| Primer Name | **Chromosome** | **Type** | **Marker** | **Primer Sequence** |
| --- | --- | --- | --- | --- |
|  |  |  |  |  |
| MTH12.M13.fp | 5 | SSR | MTH12 | tgtaaaacgacggccagtGTAAAATTTTCTATTGCA |
| MTH12.rp | 5 | SSR | MTH12 | ATGTCCTCCTGTTCTGTCCA |
| JV65/66.M13.fp | 5 | SSR | JV65/66 | tgtaaaacgacggccagtTTTCTTTCACGGTTTCTAACTTTT |
| JV65/66.rp | 5 | SSR | JV65/66 | TCATCTTCCCTTTAATTCTGATGA |
| NGA129.M13.fp | 5 | SSR | NGA129 | tgtaaaacgacggccagtCACACTGAAGATGGTCTTGAGG |
| NGA129.rp | 5 | SSR | NGA129 | TCAGGAGGAACTAAAGTGAGGG |
| At5g17.60.M13.FP | 5 | SNP | At5G17.6 | TGTAAAACGACGGCCAGTcaaatacgtgatacggtc |
| At5g17.60.col.rp | 5 | SNP | At5G17.6 | CGGTTTCTGGTTATCTAATCAGTTGAG |
| At5g17.60.C24.rp | 5 | SNP | At5G17.6 | CTACTACGGTTTCTGGTTATCTAATCAGTTGTC |
| At5g16.26.M13.FP | 5 | SNP | At5G16.26 | TGTAAAACGACGGCCAGTcagaaaatgaagtaacg |
| At5g16.26.col.rp | 5 | SNP | At5G16.26 | CAATTAAAGTAACTTGTGTGTACGG |
| At5g16.26.C24.rp | 5 | SNP | At5G16.26 | CAGTTCAATTAAAGTAACTTGTGTGTACGT |
| At5g37710.M13.fp | 5 | SNP | At5G37710 | tgtaaaacgacggccagtGATGGCGGTTTTGGTTG |
| At5g37710.col.rp | 5 | SNP | At5G37710 | CTAACATTGAGATCATTAAAACATGGTTAAATCT |
| At5g37710.c24.rp | 5 | SNP | At5G37710 | CCGAGATCATTAAAACATGGTTAAATCC |
| ATHPHYC.M13.fp | 5 | SSR | ATHPHYC | tgtaaaacgacggccagtCTCAGAGAATTCCCAGAAAAATCT |
| ATHPHYC.rp | 5 | SSR | ATHPHYC | AAACTCGAGAGTTTTGTCTAGATC |
| S0262.M13.fp | 5 | SSR | S0262 | tgtaaaacgacggccagtATCATCTGCCCATGGTTTTT |
| S0262.rp | 5 | SSR | S0262 | TTGCTTTTTGGTTATATTCGGA |
| NGA139.M13.fp | 5 | SSR | NGA139 | tgtaaaacgacggccagtGGTTTCGTTTCACTATCCAGG |
| NGA139.rp | 5 | SSR | NGA139 | AGAGCTACCAGATCCGATGG |
| ciw5.M13.fp | 4 | SSR | ciw5 | tgtaaaacgacggccagtGGTTAAAAATTAGGGTTACGA |
| ciw5.rp | 4 | SSR | ciw5 | AGATTTACGTGGAAGCAAT |
| At3G63470.M13.fp | 3 | SNP | At3G63470 | tgtaaaacgacggccagtCCGTCAACGAATCCGC |
| At3G63470.col.rp | 3 | SNP | At3G63470 | GTGCCCCTAACTAAAGATGCAAACCA**C**A |
|  |  |  |  |  |
|  |  |  |  |  |
| Primer Name | **Chromosome** | **Type** | **Marker** | **Primer Sequence** |
|  |  |  |  |  |
| At3G63470.C24.rp | 3 | SNP | At3G63470 | CTAACTAAAGATGCAAACCA**C**G |
| NGA112.M13.fp | 3 | SSR | NGA112 | tgtaaaacgacggccagtCTCTCCACCTCCTCCAGTACC |
| NGA112.rp | 3 | SSR | NGA112 | TAATCACGTGTATGCAGCTGC |
| FUS6.2.M13.fp | 3 | SSR | FUS6.2 | tgtaaaacgacggccagtTTCCTTGATCAGATTTGGTCG |
| FUS6.2.rp | 3 | SSR | FUS6.2 | TCGTTACACTGGCTTGCTTG |
| ciw27.M13.fp | 3 | SSR | ciw27 | tgtaaaacgacggccagtTAACTTCCTTGGGCAAAC |
| ciw27.rp | 3 | SSR | ciw27 | GCGGTATGAAACACAACTTA |
| CDC2BG.M13.fp | 3 | SSR | CDC2BG | tgtaaaacgacggccagtATTGAACTGTGTTGGTTTCTGG |
| CDC2BG.rp | 3 | SSR | CDC2BG | GGGAAAAACGAAGTGACGTG |
| At3G19.53.M13.fp | 3 | SSR | At3G19.53 | tgtaaaacgacggccagtCCTGAGGAGATTCTGGAG |
| At3G19.53.rp | 3 | SSR | At3G19.53 | CTTCCGTTGCAACAGCATCC |
| ciw4.M13.fp | 3 | SSR | ciw4 | tgtaaaacgacggccagtGTTCATTAAACTTGCGTGTGT |
| ciw4.rp | 3 | SSR | ciw4 | TACGGTCAGATTGAGTGATTC |
| F1P2TGF.M13.fp | 3 | SSR | F1P2-TGF | tgtaaaacgacggccagtTTTGTCTGAAGATGTGGAGAGAGAG |
| F1P2TGF.rp | 3 | SSR | F1P2-TGF | CAAAACCCCACTCTTCATTATTGTT |
| NF20D22.M13.fp | 1 | SSR | NF20D22 | tgtaaaacgacggccagtAACAAAATGAGTTTCTCTGCATG |
| NF20D22.rp | 1 | SSR | NF20D22 | CCCAAGTGACGTCTGGTTTC |
| NF7G19.M13.fp | 1 | SSR | NF7G19 | tgtaaaacgacggccagtTCGTTGAAAACGATTAGATTGG |
| NF7G19.rp | 1 | SSR | NF7G19 | TTCAAAAATCGTGAGATGAAATG |
| JV28/29.M13.fp | 1 | SSR | JV28/29 | tgtaaaacgacggccagtCACTTGTTCTGAAACGAAATTGA |
| JV28/29.rp | 1 | SSR | JV28/29 | GCTTCTCATTGCACTCCTTTG |
| NF19G9.M13.fp | 1 | SSR | NF19G9 | tgtaaaacgacggccagtAAGAACTTAATTTCTCTCACCCG |
| NF19G9.rp | 1 | SSR | NF19G9 | AGTTGGTCCTCGAGCTCTCC |
| NF21J9.M13.fp | 1 | SSR | NF21J9 | tgtaaaacgacggccagtGAGACGAAGAAGATGGATTCTG |
| NF21J9.rp | 1 | SSR | NF21J9 | CAGATTTCTTGCCAAGTTTCATC |
| ciw12.M13.fp | 1 | SSR | ciw12 | tgtaaaacgacggccagtAGGTTTTATTGCTTTTCACA |
| ciw12.rp | 1 | SSR | ciw12 | CTTTCAAAAGCACATCACA |
|  |  |  |  |  |
| Primer Name | **Chromosome** | **Type** | **Marker** | **Primer Sequence** |
|  |  |  |  |  |
| At3G59850intron2fp | 3 | INDEL | intron2 | ATCGAGAATGTCGCTTGTG |
| At3G59850intron2rp | 3 | INDEL | intron2 | gttctctctttgtagttgtgttg |
| NGA112.fp | 3 | SSR | NGA112 | CTCTCCACCTCCTCCAGTACC |
| NGA112.rp | 3 | SSR | NGA112 | TAATCACGTGTATGCAGCTGC |
| FUS6.2.fp | 3 | SSR | FUS6.2 | TTCCTTGATCAGATTTGGTCG |
| FUS6.2.rp | 3 | SSR | FUS6.2 | TCGTTACACTGGCTTGCTTG |
| At3g60440.fp | 3 | SNP | 60440 | CAATCACTTCCAACGTAGTCTC |
| At3g60440.rp | 3 | SNP | 60440 | ATCGATTGAAGTGACGTC |
| At3G60620f | 3 | SNP | 60620 | CGTTCGTAAGACTTCATGG |
| At3G60620r | 3 | SNP | 60620 | catgtctttcctcttcccaatc |
| At3G60730f | 3 | SNP | 60730 | gacttgagaggttccattc |
| At3G60730r | 3 | SNP | 60730 | GTGACTTTTACAAACGTGGAC |
| At3g60860fp | 3 | SNP | 60860 | GTTTTACTCCAATGGTGCTG |
| At3g60860rp | 3 | SNP | 60860 | ctatacacttgttccacgg |
