# Supplementary material for: Independent S-Locus Mutations Caused Self-Fertility in Arabidopsis thaliana
Source: PLoS Genet. 2009 Mar 20;5(3):e1000426. doi: 10.1371/journal.pgen.1000426 (PMC2650789; doi:10.1371/journal.pgen.1000426)
Supplement: Table S2 — Fine mapping using NIL3.2 plants. (0.04 MB DOC) [file pgen.1000426.s003.doc]

**Table S2. Fine mapping using NIL3.2 plants.**

| **NIL3.2 plant ^a^** | **F2 ^b^** | **F3 ^b^** | **intron2 ^c^** | **60440 ^c^** | **60620 ^c^** | **60730 ^c^** | **60860 ^c^** | **FUS6.2 ^c^** | **NGA112 ^c^** |
| --- | --- | --- | --- | --- | --- | --- | --- | --- | --- |
|  |  |  |  |  |  |  |  |  |  |
| 6.7c ^d^ | SC | SC/SI mix | 2 | 2🡪 | 1 |  | 1 | 1 | 1 |
| 5.3f | SC | SI | 2 | 2 | 2 | 2 | 1 | 1 | 1 |
| 11.6e | SC | SI | 2 | 2 | 2 | 2 | 1 | 1 | 1 |
| 12.4g | SC | SI | 2 | 2 | 1 |  | 1 | 1 | 1 |
| 8.8d | SC |  | 2 | 1 |  |  |  | 1 | 1 |
| 7.8h | SC |  | 2 | 1 |  |  |  | 1 | 1 |
| 8.2e | SC | SI | 2 | 2 | 2 | 1 | 1 | 1 | 1 |
| 7.9g ^d^ | SC | SC/SI mix | 1 | 1 | 1 | 🡨2 | 2 | 2 | 2 |
| 8.2b | SC | SI | 1 | 1 | 2 | 2 | 2 | 2 | 2 |
| 4.4c ^d^ | SC | SI/SC mix | 1 | 1 |  |  | 1 | 🡨2 | 2 |
| 11.10e | SC | SI | 3 | 3 | 3 |  | 2 | 2 | 2 |
| 14.1g | SC |  | 3 | 2 |  |  |  | 2 | 2 |

^a^ Individual recombinant NIL3.2 F2s are listed.

**^b^** Pollination phenotype observed in the F2 and F3 generations. SC: self-compatible; SI: self-incompatible.

**^c^** F2 genotypes for each of the indicated markers are shown: “1” indicates homozygosity for the Col-0 allele, “2” indicating heterozygosity (Col-0/C24), and “3” indicating homozygosity for the C24 allele.

^d^ Informative NIL3.2 F2s used for mapping. The direction of the arrows in these rows point towards the inferred position of QTL3.2. As described in the text, NIL3.2 F2 SI plants were ignored for fine mapping because the SI trait can be erased from one generation to the next (e.g. see family 12.4g). Rescreening the SC phenotype in the F3 generation is important to validate the presence of the target gene within the recombinant region. Based on this criterion, the three informative families place QTL3.2 between markers 60440 and 60730.
