# Supplementary material for: Independent S-Locus Mutations Caused Self-Fertility in Arabidopsis thaliana
Source: PLoS Genet. 2009 Mar 20;5(3):e1000426. doi: 10.1371/journal.pgen.1000426 (PMC2650789; doi:10.1371/journal.pgen.1000426)
Supplement: Table S3 — Pollination phenotypes in two NIL3.2 F3 families that segregated for self-compatibility. (0.04 MB DOC) [file pgen.1000426.s004.doc]

**Table S3. Pollination phenotypes in two NIL3.2 F3 families that segregated for self-compatibility ^a^**

**Family 6.7c Family 7.9g**

| **individual** | **intron2 ^a^** | **NGA112 ^a^** | **phenotype ^b^** |  | **individual** | **intron2 ^a^** | **NGA112 ^a^** | **phenotype ^b^** |
| --- | --- | --- | --- | --- | --- | --- | --- | --- |
| E10 | 2 | 1 | SC |  | E1 | 1 | 2 | PSC |
| E11 | 3 | 1 |  |  | E2 | 1 | 3 | SI |
| E12 | 3 | 1 | SI |  | E3 | 1 | 1 | SC |
| F10 | 2 | 1 | SI |  | E4 | 1 | 2 | SC |
| F11 | 3 | 1 | PSC |  | E5 | 1 | 2 | PSC |
| F12 | 3 | 1 | SC |  | E6 | 1 | 3 | SI |
| G10 | 2 | 1 | SC |  | F2 | 1 | 3 | SC |
| G12 | 2 | 1 | SC |  | F3 | 1 | 1 | SC |
| H10 | 3 | 1 | SC |  | G2 | 1 | 2 | SI |
| H11 |  | 1 | SC |  | G3 | 1 | 1 | SC |
| H12 | 2 | 1 | SC |  | G4 | 1 | 1 | SC |
|  |  |  |  |  | G5 | 1 | 2 | PSC |
|  |  |  |  |  | G6 | 1 | 2 | SC |

^a^ Numbers in the columns as in Table S2.

**^b^** SC:self-compatible; PSC: partially self-compatible; SI: self-incompatible. Note the lack of tight correlation between phenotype and genotype.
